# Universal suprapubic approach for complete mesocolic excision and central vascular ligation using the da Vinci Xi^®^ system: from cadaveric models to clinical cases

**DOI:** 10.1007/s11701-016-0664-y

**Published:** 2017-02-01

**Authors:** Shen Ann Yeo, Gyoung Tae Noh, Jeong Hee Han, Chinock Cheong, Hubert Stein, Amy Kerdok, Byung Soh Min

**Affiliations:** 1 Department of Colorectal Surgery, Singapore General Hospital, Outram Road, Singapore, 169608 Singapore; 2 Department of Surgery, Yonsei University College of Medicine, 50-1 Yonsei-ro, Seodaemun-gu, Seoul, 120-752 Korea; 3 Department of Clinical Development Engineering, Intuitive Surgical, 1020 Kifer Road, Sunnyvale, CA 94086-5304 USA

**Keywords:** Robotic surgery, da Vinci Xi, Right hemicolectomy, Left hemicolectomy, Complete mesocolic excision, Suprapubic

## Abstract

There has been little enthusiasm for performing robotic colectomy for colon cancer in recent years due to multiple factors, one being that the previous robotic systems such as the da Vinci Si^®^ (dVSi) were poorly designed for multi-quadrant surgery. The new da Vinci Xi^®^ (dVXi) system enables colectomy with central mesocolic excision to be performed easily in a single docking procedure. We developed a universal port placement strategy to allow right and left hemicolectomies to be performed via a suprapubic approach and a Pfannensteil extraction site. This proof of concept paper describes the development and subsequent clinical application of this setup. After extensive training on the dVXi system concepts in collaboration with clinical development engineers, we developed a port placement strategy which was tested and adapted after performing experimental surgery in three cadaveric models. Subsequently our port placement was used for two clinical cases of suprapubic right and left hemicolectomy. With some modifications of port placements after the initial cadaveric colectomies, we have developed a potentially universal suprapubic port placement strategy for robotic colectomy with complete mesocolic excision and central vascular ligation using the dVXi robotic system. This port placement strategy was applied successfully in our first two clinical cases. Based on our cadaveric laboratory as well as our initial clinical application, the suprapubic port placement strategy for the dVXi system with its improved features over the dVSi can feasibly perform right and left hemicolectomy with complete mesocolic excision and central vascular ligation. Further studies will be required to establish efficacy as well as safety profile of these procedures.

## Introduction

Complete mesocolic excision (CME) with central vascular ligation (CVL) is a recently described concept proposed for the standardized radical resection for colon cancer [1]. It has similar principles to the total mesorectal excision (TME) technique described by Heald [2] in that it emphasizes en-bloc removal of the entire potential cancer-bearing package of the mesocolon along with the draining lymphatic tissue enveloped in its embryonic fascial layers all the way up to the origin of the primary feeding vessels to ensure radial lymph node dissection. Despite there being various controversies regarding the details of the technique itself, the concept has been shown to have good oncological outcomes [3] and is gradually gaining recognition.

At this moment in time, the robotic TME procedure is steadily gaining popularity worldwide. However, unlike robotic TME, which has standardized robotic port and patient position setups using the previous generation of the robotic system—the da Vinci Si^®^ (dVSi) [4–6], robotic assisted colectomy still remains unpopular. The reason for this is twofold: (1) besides the fact that there is little evidence supporting the use for the robotic system in performing colectomies for cancer, (2) the dVSi system was not optimized for colectomies due to its physical configuration and design. Particularly, the limited range of the robotic instruments as well as the inability of the dVSi robotic system to perform surgery over a large area within the abdomen resulted in a major obstacle for the development of robotic surgical procedures such as colectomies where the surgical workspace is large and multi-quadrant in nature.

With the introduction of the recent da Vinci Xi^®^ (dVXi) system, multiple technological advances have been made over the previous generation dVSi, which optimizes the robotic system to be able to perform multi-quadrant surgical procedures. Given this improvement, the authors were inspired to develop and establish a universal setting for robotic colectomies. The purpose of this proof of concept study is to describe and report a standardized suprapubic port setup with Pfannenstiel extraction site for robotic right and left hemicolectomy using the dVXi system. Technical feasibility of this setup was first established by performing the procedures on cadaveric models and subsequently applied to clinical cases.

## Methods

### Cadaver lab study

A proof of concept study was performed using a total of three fresh cadaver models in the cadaveric laboratory at Intuitive Surgical^®^ in Sunnyvale, CA, USA. Two Caucasian males and one Caucasian female cadaver were used. All three cadavers had no prior known intraabdominal disease or surgical history. A standardized suprapubic port setup was used for all procedures, and both the internal and external views of the procedures were recorded and reviewed and are described below.

## Cadaveric suprapubic right hemicolectomy (SRHC)

### Port placement and robotic setup

**Fig. 1:**
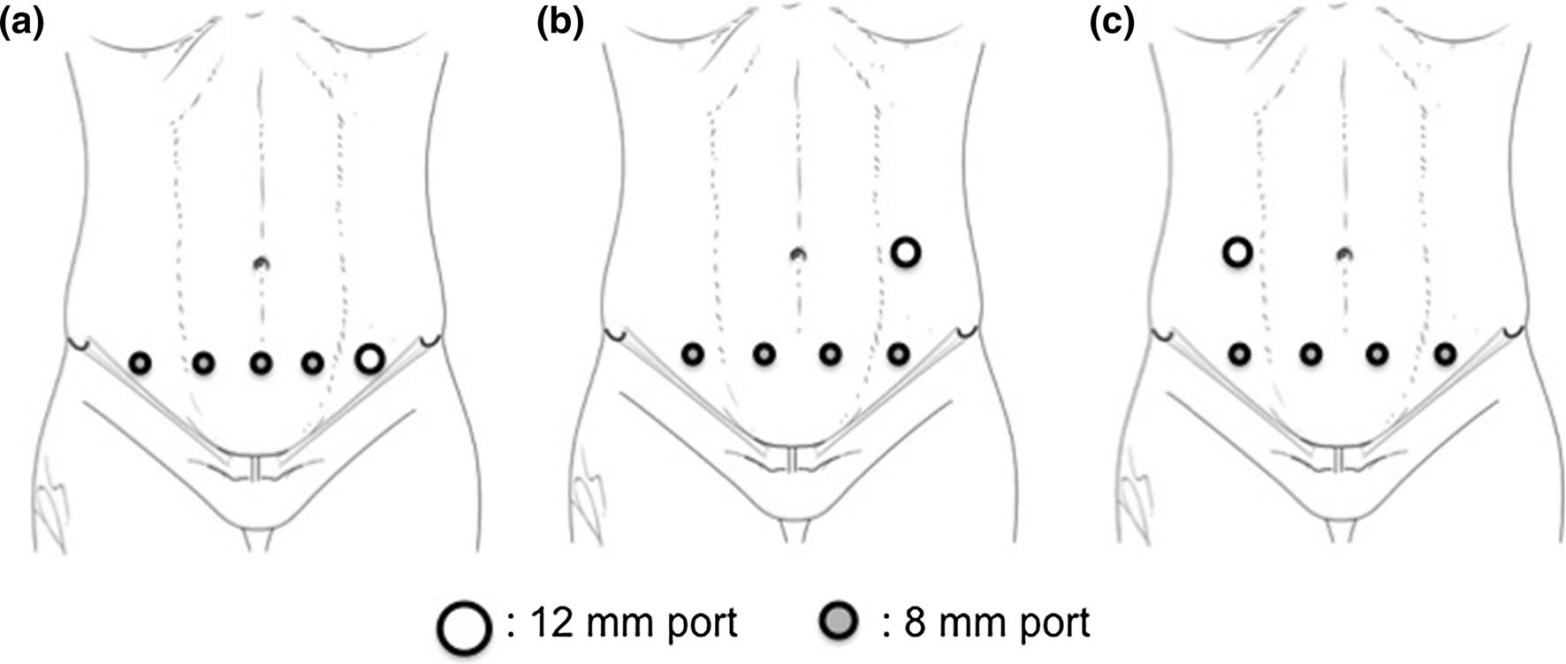
Port placement for dVXi suprapubic colectomy **a** initial port placement for SRHC, **b** adapted port placement for SRHC, **c** port placement for SLHC

The patient was placed in a lithotomy position and in 15° of Trendenlenburg and left tilt. Four 8 mm robotic trocars as well as a 12 mm laparoscopic trocar were place sequentially in a transverse line, 3–4 cm above the pubic symphysis (Fig. 1a). Both the lateral trocars were placed a minimum of 3 cm medial to the anterior superior iliac spine on each side, and the distance between each trocar along the line was evenly distributed, with a recommended distance of 6–8 cm in between each trocar. The robotic cart was docked from the right side of the patient. A subsequent modification of the port placement for the second cadaveric case was made, with the four robotic trocars to be placed on the suprapubic line and the 12 mm laparoscopic trocar on the left midclavicular line at the level of the umbilicus (Fig. 1b). This was to allow further distance between the robotic trocars along the suprapubic line, and also enable easier access for the bedside surgical assistant during the procedure as the robotic arms would frequently interfere with the assistant in the prior port configuration. The robotic system was docked from the right side of the patient and targeted at the hepatic flexure prior to commencement of surgery.

### Surgical procedure for right hemicolectomy

**Fig. 2:**
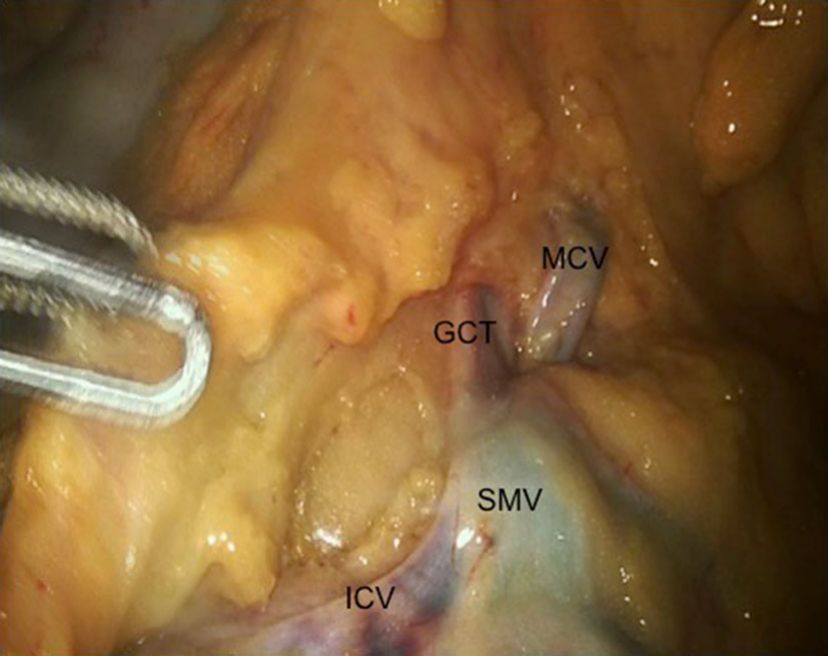
Cadaveric SRHC showing venous anatomy. *SMV* superior mesenteric vein, *MCV* middle colic vein, *GCT* gastrocolic trunk, *ICV* ileocolic vein

Mobilization of the right colon and mesocolon was first performed via an inferior approach. The peritoneal reflection of the small bowel mesentery was incised and dissection into the surgical place above Gerota’s fascia was performed to mobilize the entire right colon mesentery from the retroperitoneal structures. The entire C loop of the duodenum as well as the pancreatic head was exposed, with the hepato-duodenal ligament excised and takedown of the hepatic flexure in a lateral to medial fashion. Subsequently lymphadenectomy was performed along the superior mesenteric vessels, with individual exposure and ligation of the ileocolic, right colic and middle colic vessels at their origin from the superior mesenteric artery and vein. The gastrocolic trunk was exposed (Fig. 2) and the colonic branches were ligated preserving the pancreatic branches. Omentectomy was performed at the level inferior to the gastro-epiploic vessels, and the transverse colon was mobilized from the omentum. Subsequently the mesocolon of the transverse colon as well as the mesentery of the ileum were divided till the desired resection margins, taking both proximal and distal resection margins with the robotic stapling device. Enterotomies were made at both the ileum and the distal transverse colon, and an intracorporeal isoperistaltic side to side anastomosis was performed using the robotic stapler, with suturing of the initial enterotomy site performed to complete the anastomosis. The specimen was extracted using a Pfannenstiel incision extending between the central two suprapubic ports.

## Cadaveric suprapubic left hemicolectomy (SLHC)

### Port placement and robotic setup

A similar configuration to the right hemicolectomy was followed for the left hemicolectomy procedure. However, in this case the patient was placed in 15° of right tilt instead. For left hemicolectomy procedure, a similar suprapubic port placement as the right hemicolectomy for four 8 mm robotic arms was applied. The 12 mm laparoscopic assistant port was inserted in the right midclavicular line at the level of the umbilicus (Fig. 1c). The robotic system was docked from the left side of the patient and targeted at the splenic flexure prior to commencement of surgery.

### Surgical procedure for left hemicolectomy

A medial to lateral approach for mobilization of the left colon was performed. The mesenteric peritoneum was incised at the level of the sacral promontory, separating the mesenteric plane of the sigmoid and descending colon from the retroperitoneal structures. Dissection proceeded cephalad to expose the root of the inferior mesenteric artery and from there further dissection was carried out to expose the left colic artery which was ligated. Mobilization carried on superiorly to the inferior mesenteric vein, bringing the pancreas down away from the mesocolon and entering the lesser sac from the inferior aspect. The inferior mesenteric vein was then ligated at the level of the lower border of the pancreas. The middle colic artery was exposed after dissection of the transverse mesocolon, and the left branch isolated and ligated. Subsequently the entire left colon was freed from the lateral parietal peritoneal attachments including the sigmoid and descending colon, with full mobilization of the splenic flexure to allow for tension free anastomosis. The colonic mesentery was divided using an energy device up to the proximal and distal margins, which were stapled off using a laparoscopic linear stapler. An intracorporeal isoperistaltic side to side anastomosis was performed using a linear stapler and sutures.

### Results of cadaveric lab hemicolectomy procedures

Operation times were 106 and 93 min for the two SRHC procedures from skin incision to completion of intracorporeal anastomoses. The SLHC procedure took a total of 87 min for completion. All the cadaveric procedures were completed successfully, with identification, exposure and individual ligation of the relevant vascular anatomy. During all the procedures, the robotic arms were actively managed by the patient-side assistant to avoid arm interferences.

## Clinical cases

Institutional Review Board approval (IRB No. 4-2016-0313) was obtained for the purposes of this study. For both the clinical cases of suprapubic right and left hemicolectomy, informed consent from both individual patients to undergo their respective procedures was obtained.

## Suprapubic right hemicolectomy

**Fig. 3:**
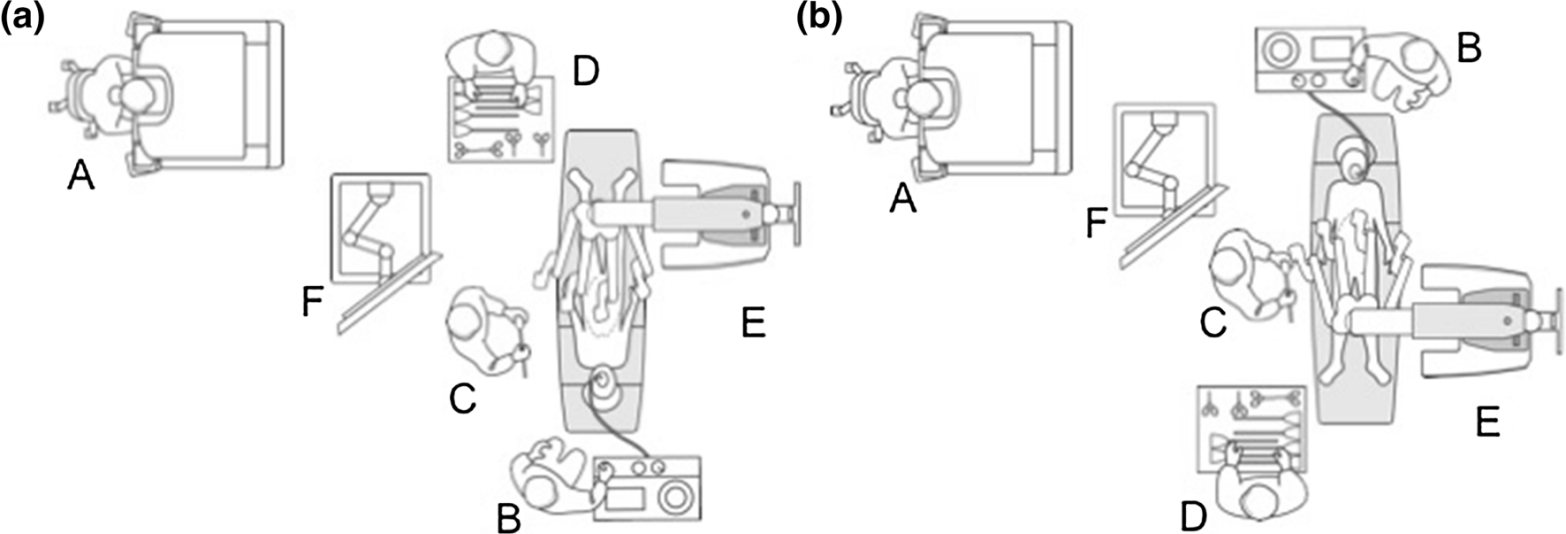
Operation theatre layout for suprapubic colectomy. **a** SRHC setup, **b** SLHC setup. *A* Surgeon console, *B* anesthetist, *C* table side assistant, *D* scrub nurse, *E* dVXi patient cart, *F* dVXi Vision cart

**Fig. 4:**
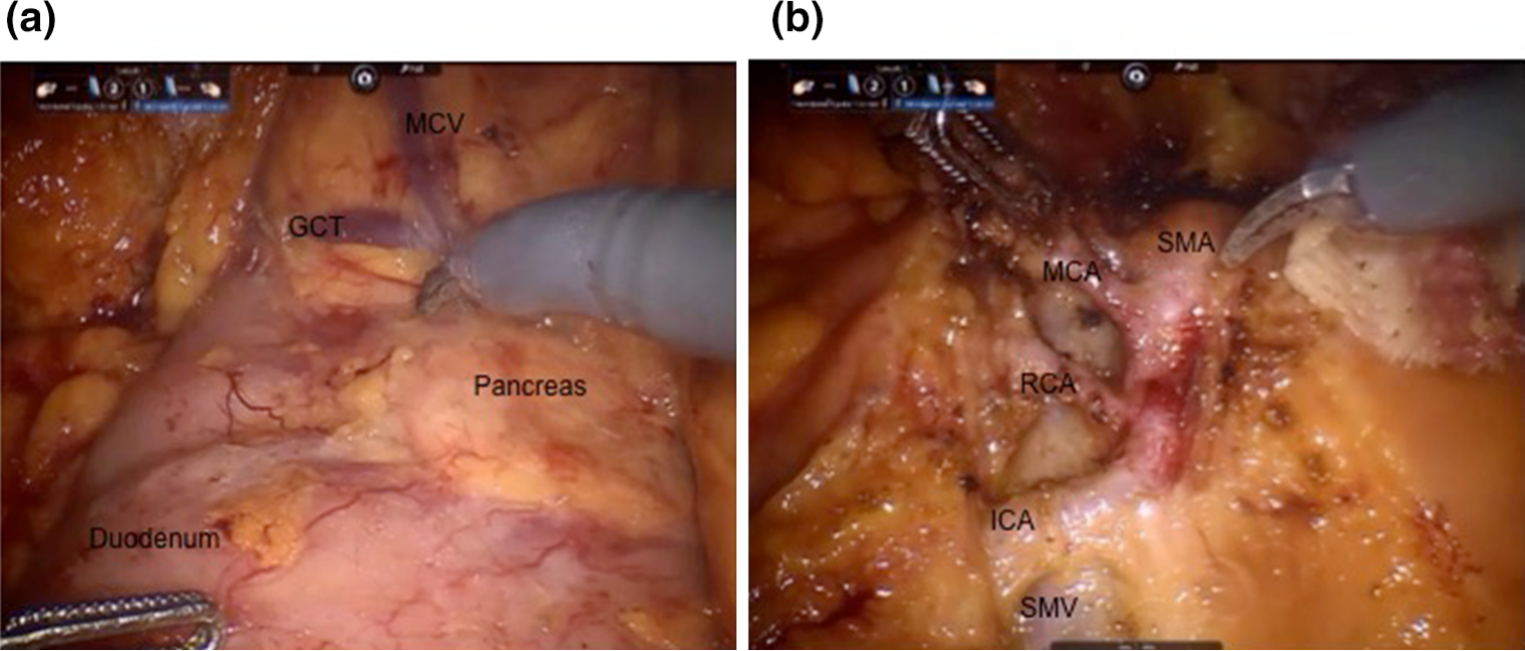
Intraoperative pictures of SRHC. **a** After inferolateral mobilisation: *GCT* gastrocolic trunk, *MCV* middle colic vein. **b** Vascular anatomy shown before ligation. *SMA* superior mesenteric artery, *MCA* middle colic artery, *RCA* right colic artery, *ICA* ileocolic artery, *SMV* superior mesenteric vein

**Fig. 5:**
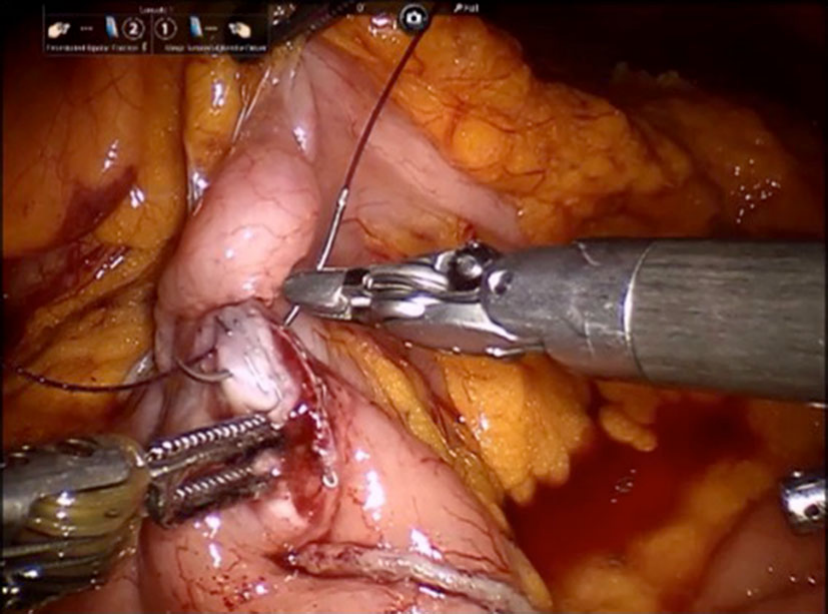
Intracorporeal anastomosis for SRHC

An 85-year-old female patient was diagnosed with a distal ascending colon cancer with no other synchronous lesions on colonoscopy and confirmed by pathological reports. She underwent a SRHC performed in the manner described in the cadaveric laboratory above. The operation theatre setup is shown in Fig. 3a. Complete mesocolic excision and central vascular ligation were performed. After infero-lateral mobilization of the terminal ileum, caecum and ascending colon exposing the duodenum and pancreas (Fig. 4a), the ileocolic, right colic and middle colic arteries were dissected and ligated at their origins from the superior mesenteric artery (Fig. 4b). The ileocolic and middle colic veins as well as the colic branches of the gastrocolic trunk were also dissected out, identified and ligated. Intracorporeal side to side anastomosis was performed as previously described (Fig. 5), and the specimen was placed in a laparoscopic pouch and extracted through a Pfannenstiel incision made between the central two suprapubic robotic ports. Operative time was 225 min and estimated blood loss was 50 ml. The patient was discharged well on the 10th postoperative day with no complications. Histology returned as moderately differentiated adenocarcinoma, pT3N2a with 4 out of 25 lymph nodes involved. Margins were clear with a 10.5 and 32.5 cm proximal and distal margin, respectively.

## Suprapubic left hemicolectomy

**Fig. 6:**
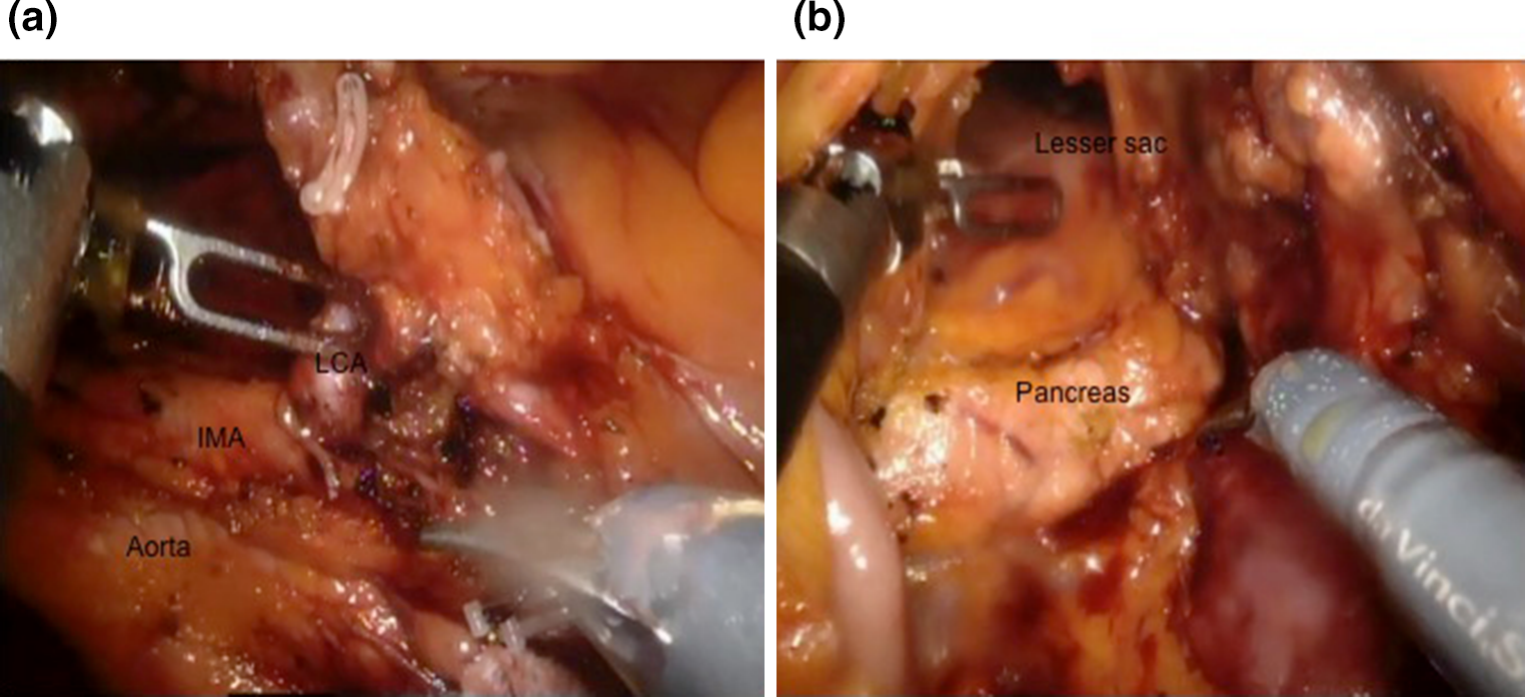
Intraoperative pictures of SLHC. **a** Dissection of left colic artery: *IMA* inferior mesenteric artery, *LCA* left colic artery. **b** Medial mobilisation of mesocolon from pancreas to enter lesser sac

A 66-year-old female patient was diagnosed with proximal descending colon cancer on colonoscopy and biopsy. She also had symptomatic gallstones prior to surgery, and a robotic SLHC along with cholecystectomy was performed. The operation theatre setup is shown in Fig. 3b. After port placement and pneumoperitoneum the gallbladder was targeted first, and after the cholecystectomy was completed, the gallbladder was placed in a laparoscopic pouch and placed in the right subphrenic space. Subsequently the robotic arms were undocked except for the camera arm, and the splenic flexure was then targeted to proceed with the left hemicolectomy. The left colic artery was ligated at its origin preserving the inferior mesenteric artery (Fig. 6a). Subsequently mobilization was performed of the mesocolon from the pancreas to enter the lesser sac medially (Fig. 6b), and laterally to completely bring down the splenic flexure. The left branch of the middle colic artery was also identified and ligated at its root, and after completion of proximal and distal transection the specimen was inserted into a separate laparoscopic pouch and both the gallbladder and colon were extracted via a suprapubic incision after performing anastomosis. Operative time was 224 min and estimated blood loss 50 ml. The patient had an uneventful postoperative course and was discharged on the 5th postoperative day. Histology returned as moderately differentiated adenocarcinoma, pT3N2a with 4 out of 22 lymph nodes involved. Margins were clear of disease with a proximal and distal margin of 9.5 and 7.3 cm, respectively.

## Discussion

The da Vinci^®^ robotic system has gained much popularity in the field of colorectal surgery over recent years, with its inherent advantages over laparoscopy such as improved 3D vision, stable retraction and camera platforms, as well as wristed instrumentation with 7° of freedom of motion [7]. However, it is currently mostly used for low anterior resection, where there is some evidence that the improved visualization as well as controlled fine dissection can give better outcomes to patients such as improved sexual and urinary function [8] as well as lower conversion rates, with comparable oncological outcomes to open and laparoscopic surgery [9].

The da Vinci robotic system has seen some changes over the years, with various different models introduced over the last few years—namely the da Vinci Standard, da Vinci S system, as well as the da Vinci Si system which was introduced in 2009. These three models of the robotic system, although improving with each version, were designed based on a similar principle of function. These systems all operate based on a patient side cart designed with a central column, from which robotic arms extend out to perform the required surgical procedure. Given this design and concept, optimal port placement that allows all arms to cover the necessary surgical field would be in an arc or semicircular configuration—with all ports equidistant from a single targeted anatomical structure, and at least a distance of 8 cm apart between ports.

As a result of this, there are some difficulties that occur due to the restrictions inherent to the design of all the previous da Vinci^®^ systems. This setup leads to problems when the surgical field is wider than a single abdominal quadrant, with issues of arm interferences and inadequate reach once the boundaries of that targeted surgical workspace is exceeded. As such, for the robotic low anterior resections, either hybrid procedures (laparoscopic splenic flexure takedown followed by robotic proctectomy) or multiple dockings of the robotic system are required usually in at least two stages—firstly for vascular ligation with colonic and splenic flexure mobilization and subsequently for pelvic dissection. This limitation has curbed the enthusiasm for surgeons in perform robotic colectomies, which frequently require a surgical workspace that traverses more than one abdominal quadrant to perform.

However, with the introduction of the new dVXi system in 2014, the technical difficulties faced by surgeons performing multi-quadrant procedures have now been decreased due to the technological advancement and new design of the robotic system. The dVXi system is currently designed with a rotatable overhead boom that extends from the central column of the patient cart, and four slimmer robotic arms extending from special FLEX joints attached to this overhead boom. With this new design come several advantages of the dVXi system over the older dVSi system. Firstly the mobility of the boom and robotic arms is increased, which allows port placement up to a minimum of 6 cm apart (compared to 8 cm in the Si). This allows port placement to be more flexible, especially in the patients with a smaller body habitus—allowing the ports to be still placed in the optimal position to perform surgery without experiencing significant arm interference. The ports are all now of the same size (8 mm), including the camera port—which allows port hopping of the camera and various arms reducing restrictions of the intraoperative view. This design also allows for a much simpler port placement, i.e., a linear port placement arrangement as the robotic arms are designed to be able to work in parallel: this simplifies port placement greatly, and also it helps to maximize the surgical workspace and minimize arm collisions intraoperatively.

Secondly, given the boom mounting of the arms, the patient cart can now be placed in a much more variable position and still be able to access the appropriate target anatomy. The base location of the patient cart is now non critical, and may be positioned at a location where no one is needed to stand, which allows easy access for the assistant during the surgery. Unlike the dVSi system where the patient cart central column, camera arm and target anatomy need to be in a linear arrangement to maximize surgical workspace, the dVXi patient cart may be placed in almost any position relative to the patient and still be able to perform the surgical procedure. This flexibility in patient cart placement allows for a consistent OT setup, regardless of procedure, and avoids needing to move the patient cart or the patient during surgery. However, it is important to note that the dVXi patient cart is still limited in that it is not designed to work at 180° from the patient base (e.g., the cart cannot be positioned on the right side of the patient for a left hemicolectomy).

Thirdly is the new concept of targeting of the robotic system. The targeting function new to the dVXi system has been designed to align the boom of the patient cart over the surgical workspace. This has a few functions, namely: (1) ensuring all the robotic arms can dock to the ports; (2) orients all the arms towards the target anatomy; as well as (3) allowing all the joints to be within the “sweet spots” to maximize the range of motion of the robotic arms.

Finally, the robotic arms in the dVXi are much slimmer compared to those in the dVSi (arm spar width 1.7′ vs 2.9′), and furthermore the robotic instruments are 5 cm longer with a wider range of movement enabled by an additional joint on the robotic arm. This results in two advantages of the dVXi system: namely that it can has an increased reach which leads to a larger surgical workspace, and also reduces arm interference during the surgery as the arms are slimmer compared to the dVSi. The relevant differences between the dVXi and dVSi system are summarized in Table 1.

**Table 1:** Differences between the dVSi and dVXi robotic systems

	da Vinci Si	da Vinci Xi	Remarks
Range of motion	Outer yaw 336° Outer pitch 149°	Outer yaw 504° Outer pitch 177°	Increased range of motion of robotic arms of dVXi
Instrument reach		1.75′ additional reach	Increase robotic arm reach of dVXi
Overhead boom rotation	No	Yes	Allows for patient cart of dVXi to be docked at any position
Targeting system	No	Yes	Allows optimization of boom positioning to minimize arm interference of dVXi
Camera arms	1	4	Allows camera to be interchanged to any of the arms on the dVXi
Arm spar width	2.9′	1.7′	Decreases rate of arm collisions during surgery

Given the abilities of the new system, we were inspired to develop a standardized port setup for colectomies, which would provide a guide for surgeons new to the dVXi robotic system in general or even for those who have predominantly performed proctectomies and are unfamiliar with robotic colectomies, so that they may take up the procedures in a more straightforward manner.

There are a few reasons why we chose to further develop this idea despite the current low level of enthusiasm in the colorectal field for robotic colectomies. Firstly, we believe that the poor uptake of robotic colectomy stems from the inability of the previous robotic systems to adequately address the problem of multiquadrant abdominal surgery. With the introduction of the new dVXi system, we believe that robotic colectomies would now be much more straightforward procedures compared to the past. Also applying this suprapubic port placement and setup would be simple and straightforward, unlike having to worry about the multiple port placements and changing of the robotic cart position in the past.

Secondly, our suprapubic port setup requires a Pfannenstiel extraction of the specimen as well an intracorporeal anastomosis to be performed. It would be near impossible to perform an extracorporeal anastomosis for a patient with a suprapubic extraction site. Performing an intracorporeal anastomosis requires the ability to perform intracorporeal suturing, for which the robotic instruments—given the dexterity of movement—would be well placed to overcome the technical challenges of laparoscopic suturing.

Thirdly is the use of a Pfannenstiel incision for specimen extraction. There are currently some recommendations of port placement strategies for the dVXi robotic colectomy procedures based on the linear port placement configuration [10]. The initially described diagonally based port placement setups for the right and left hemicolectomies are certainly feasible and will allow the procedure to be performed in a satisfactory manner; however, in these cases the extraction port site would have to be sited at the umbilical port area, and with this extraction site, cosmesis would be less desirable as the port scars would be easily visible. Furthermore, the umbilical wound site has been reported to have the highest risk of hernia development, compared to other incision sites. As such, we had come up with the idea of port placement at the suprapubic area, which would allow us to circumvent these problems of the previously recommended setup: (1) cosmesis would be improved as most of the ports save one will be hidden in the suprapubic area, and (2) the extraction site can now be via a Pfannenstiel incision which has been shown to be superior to an umbilical incision with regards to lower rates of hernia formation [11–13] as well as reduced postoperative pain, with consequently quicker discharge from hospital and return to work. The suprapubic port configuration is now viable because the minimum distance between ports for the Xi system has been reduced from 8 cm (on the Si system) to 6 cm, allowing all four robotic ports to be placed in the suprapubic area. An 8 cm distance between the robotic ports in a linear suprapubic configuration would likely be almost impossible to achieve in a patient of average sized body habitus due to the distance between both anterior superior iliac spines, except in patients with much larger body habitus, which would have limited the generalizability of this standardization.

Finally, the concept of CME with CVL for colectomy has been shown to be potentially superior to the conventional technique, with increased lymph node yield, better proximal and distal margins [14] as well as improved oncological outcomes in certain patient groups [15]; with no added complication risks if performed by trained practitioners. There are certainly technical challenges in performing CME and CVL as it requires fine meticulous dissection, identification of main arterial and venous structures and also ligation of the vessels at the root. The use of the robot, with its advantages over laparoscopy with regards to instrument dexterity and vision, may help surgeons perform a better CME and CVL with less blood loss and potentially even shorter operating times. This would be even more important in the ever increasing obese patient population with high amounts of visceral fat, which even the experienced laparoscopic surgeon may find challenging to deal with intraoperatively.

We would like to highlight that the development of this port placement was approached with much consideration and thought, as we were aware of the potential calamitous situations that could arise if these ideas were not verified before putting them to use in real patients. Thus, in the process of development of this setup, we had many sessions of discussions with the Intuitive Surgical engineers, with regards to optimal port placement sites as well as potential difficulties that we could have faced and how we could prevent problems in each of those scenarios. After a comprehensive discussion we had initially settled on a five port suprapubic placement format, which after bringing the concept to the laboratory and attempting the procedure on the first cadaver, we had adapted to putting the assistant port in the flank area of the patient so as to allow for more space between the robotic arms in the suprapubic area, to reduce the incidence of arm interference. Furthermore the assistant having to assist from the suprapubic area with the robotic arms in the way would suffer from a difficult ergonomic situation where clashing of the assisting instruments as well as potential interference of the robotic arms with the assistant himself may often occur. With the assistant approaching from the flank of the patient, we managed to reduce this issue to a minimum during our final two procedures.

Also, although the capabilities of the dVXi system allows docking to be performed from either side of the patient due to the rotating boom mechanism, we have proposed a right sided docking for SRHC and left sided docking for SLHC—this is because of the fact that the bedside assistant will need to have adequate space to allow him to assist ergonomically. If we had docked the robot on the opposite side of the tumor location, it would be unnecessarily challenging for the assistant as the robotic cart would be physically obstructing the assistant and preventing him from rendering effective assistance during surgery.

Possible barriers to adopting this suprapubic approach with the dVXi may be the unfamiliar anatomy visualization from an inferior viewpoint versus the more conventional lateral view, which also applies for the dissection from inferior to superior versus a medial to lateral approach. All cases went well with regards to the different surgical views or the different direction of dissection. We believe that both challenges might be only present initially and be easily overcome by a learning curve of just a few cases.

Performing the surgical procedures first on cadaveric models enabled us to test our ideas and ensure feasibility and safety as well as perform any troubleshooting before bringing our technique to clinical practice. As a result, surgery proceeded successfully on our patients; both had an uncomplicated convalescence and were discharged home with no complications. We plan to continue performing this procedure so that more of our patients may benefit from the advantages that it confers to recovery.

## Conclusion

Based on our cadaveric laboratory as well as our initial application into clinical practice, this suprapubic port placement strategy for the dVXi system can feasibly perform SRHC and SLHC with complete mesocolic excision and central vascular ligation, which was previously impossible using the dVSi system. The suprapubic port advantages include improved cosmesis, reduced pain and decreased incisional hernia rates from a Pfannenstiel extraction site. Further study will be required to establish safety and efficacy profile of this setup. This paper aims to be a stepping stone for standardization of dVXi robotic colectomies to allow more widespread application of these procedures.

## References

[1] Hohenberger W, Weber K, Matzel K, Papadopoulos T, Merkel S (2009). Standardized surgery for colonic cancer: complete mesocolic excision and central ligation—technical notes and outcome. Colorectal Dis.

[2] Heald RJ, Ryall RD (1986). Recurrence and survival after total mesorectal excision. Lancet.

[3] Cho MS, Baek SJ, Hur H, Min BS, Baik SH, Kim NK (2015). Modified complete mesocolic excision with central vascular ligation for the treatment of right sided colon cancer: long term outcomes and prognostic factors. Ann Surg.

[4] Kim SH (2016) Da Vinci^®^ low anterior resection procedure guide. http://www.davincisurgery.com/

[5] Min BS (2016) Da Vinci^®^ low anterior resection dual docking procedure guide. http://www.davincisurgery.com/

[6] Choi GS (2016) Da Vinci^®^ low anterior resection hybrid technique. http://www.davincisurgery.com/

[7] Lanfranco AR, Castellanos AE, Desai JP, Meyers WC (2004). Robotic surgery, a current perspective. Ann Surg.

[8] D’Annibale A, Pernazza G, Monsellato I, Pende V, Lucandri G, Mazzocchi P, Alfano G (2013). Total mesorectal excision: a comparison of oncological and functional outcomes between robotic and laparoscopic surgery for rectal cancer. Surg Endosc.

[9] Xiong B, Ma L, Huang W, Zhao Q, Cheng Y, Liu J (2015). Robotic versus laparoscopic total mesorectal excision for rectal cancer: a meta-analysis of eight studies. J Gastrointest Surg.

[10] Intuitive Surgical (2016) Da Vinci^®^ Xi surgical system. Procedure guide for LAR. http://www.davincisurgery.com/

[11] Lee L, Mappin-Kasirer B, Sender Liberman A, Stein B, Charlebois P, Vassiliou M, Fried GM, Feldman LS (2012). High incidence of symptomatic incisional hernia after midline extraction in laparoscopic colon resection. Surg Endosc.

[12] Singh R, Omiccioli A, Hegge S, McKinley C (2008). Does the extraction-site location in laparoscopic colorectal surgery have an impact on incisional hernia rates?. Surg Endosc.

[13] DeSouza A, Domajnko B, Park J (2011). Incisional hernia, midline versus low transverse incision: what is the ideal incision for specimen extraction and hand assisted laparoscopy?. Surg Endosc.

[14] Kontovounisios C, Kinross J, Tan E, Brown G, Rasheed S, Tekkis P (2015). Complete mesocolic excision in colorectal cancer: a systematic review. Colorectal Dis.

[15] Galizia G, Lieto E, De Vita F, Ferraraccio F, Zamboli A, Mabilia A, Auricchio A, Castellano P, Napolitano V, Orditura M (2014). Is complete mesocolic excision with central vascular ligation safe and effective in the surgical treatment of right sided colon cancers? A prospective study. Int J Colorectal Dis.

